# Editorial

**Published:** 1875-07

**Authors:** 


### Editorial.

### SALICYLIC ACID.

This agent is being tested by quite a number of dentists, all of whom, so far as we have had an expression, report very favorably of it. We have been using it for more than a month, and are better pleased with it, as we become more familiar with its use. It seems to serve well many of the purposes, at least, for which creosote and carbolic acid has so generally been used. The following cases represent what seems to be almost its average efficiency: Dr. T. had a left central incisor, with a medium cavity of decay upon the distal proximal surface, which had been filled for over twenty years. The pulp was never exposed. About nine years ago, it became devitalized from some unknown cause; an opening was made at that time through the palatine surface into the pulp canal and the dead pulp and debris were removed; disease of the periosteum with tendency to the formation of abscess occurred previous to this; though the ordinary treatment for such cases was employed, a chronic irritation and soreness remained, so that slight percussion on the tooth would give pain, varying somewhat at different times, from a variation of systemic and other conditions. The canal at and near the end of the root was filled with a small pledget of cotton moistened with creosote and the remainder of thc canal and chamber was filled with Hill's stopping, this remained till about one month ago, when the cotton and stopping was all removed, the latter in a very offensive condition; the opening was made through the end of the root, through which blood and pus of a very offensive character escaped. The whole was cleaned out as thoroughly as possible with the excavator and water. Then the canal was filled to one half its length with salicylic acid in crystals, and the remainder filled with Hill's stopping, Within twenty-four hours the indications of an abscess had entirely gone, the soreness had entirely abated, and has not in the least returned. This was a result that no one would have anticipated by any ordinary mode of treatment.

Mr. M. had an alveolar abscess from the second right superior molar with free and copious discharge of pus throngh the posterior buccal root; had been discharging for about five months, had been treated with carbolic acid for two months without any apparent improvement. About two weeks ago cleaned out, and injected a solution of salicylic acid and phosphate of soda in water, three times within a week, keeping it closed up in the mean time during which time the discharge of pus was entirely arrested. Before this treatment there was considerable soreness of the tooth, which had existed in varying degrees for months; after the first application this was wholly relieved, and has remained so since. Every indication is present that a permanent cure is about established. Other cases have been treated, giving about the same results, some of which we will perhaps give after more experience.

It may be used in various methods and different solutions. It may be used in the crystal, as in the first case above dedescribed, or in the watery solution, though it is very sparingly soluble in cold water, about one of the crystal to three to five hundred parts of water; warm or hot water dissolves a far larger proportion, but precipitation takes place on cooling. Other solvents are preferable when a stronger solution is required or desirable. Alcohol dissolves a large amount of the crystal; compared with water, this solution may be dilutted very considerably with water, without precipitation taking place if gentle heat is applied. Sulphuric ether dissolves a far larger proportion of the acid than alcohol. This solution is efficient when it is desirable to carry the crystal to points difficult of access, the ether immediately evaporates leaving the crystals where they have been carried in the solution.

A solution of phosphate of soda is a valuable solvent, it dissolves a large per cent of the acid, and from the promptness of its action, there is some reason to think that its operation is energized by the power ofthe phosphate of soda. Further experience, however, must verify this statement before we will venture to emphathize it.

As a disinfectant and antiseptic, it will be a very desirable ingredient for tooth pastes, powders and washes. We have in use a tooth wash prepared by Dr. Wm. Martin, of A. B. Merriam & Co., of this city, which contains salicylic acid. It a very pleasant and a very affective wash, for disinfection, we are so impressed with the value and importance of this agent that we do not hesitate to say to all, try it.

### CELLULOID.

We have received the following from the Celluloid Manufacturing Co., Newark, N. Y.:

DEAR SIR:—We have received numerous letters from members of the dental profession, informing us that Josiah Bacon, or his agents, seek to interfere with the use of Celluloid as a base for Artificial Teeth, by asserting that such use is an infringement of the Cummings patent, and by threats of prosecution against all the dentists who use Celluloid and refrain from taking out a license from the Goodyear Dental Vulcanite Company.

We caution the profession against being intimidated or deceived by such statements. The use of Celluloid is not an infringement of the Cummings patent; and as for the threatened prosecution, we guarantee protection to all dentists in the use of Celluloid against any legal assault that may be made against them on that account.

The following opinion by our counsel, HENRY BALDWIN, Jr., Esq., warrants us in publishing this guarantee, and we have no doubt will satisfy all who read it that the statement above referred to, made in the interest of rubber licenses, are false and fraudulent. Yours Truly,

CELLULOID MFG. COMPANY.

217 S. SIXTH ST., PHILADELPHIA, June 1, 1875.

*The Celluloid Manufacturing Co.:*
Newark, N. Y.

GENTLEMEN—You submit to me the following letter, viz: "Goodyear Dental Vulcanite Company, Josiah Bacon, Treasurer.

"BOSTON, FEBRUARY 15th, 1875.

"*Dr. S. H. Wirts, Steels Mills, Ills.*

"DEAR SIR:—Yours of the 12th has may attention. Permit me to inform you that the "Smith case" to which you so aptly allude, has been decided in my favor, and you will find the Supreme Court of Washington will but *confirm* that decision.

"Permit me also to inform you that the use of Celluloid is an infringement of our Patent, it being the "equivalent" of Rubber, as stated in our Patent, copy of which I enclose.

Yours truly,

"(Signed) JOSIAH BACON, TREAS."

And you ask my opinion as to the truth of the assertion made in the last paragraph "that the use of Celluloid is an infringement" of the Patent indicated.

I understand the Patent referred to as being the Reissue No. 1,904 dated March 21st, 1865, and known as the Cummings patent, that being the only patent involved in the Smith case.

In that case the testimony of the Goodyear Dental Vulcanite Company's expert, Mr. Henry B. Renwick, was that "in order to get a Cummings set of teeth, you must first have a vulcanizable compound of gum and sulphur, and finally vulcanite." The arguments of their counsel accepted this definition of what is embraced in the Reissue No. 1,904 and the opinion of the Court adopts this construction of the Cummings patent.

The claim of the patent is "the plate or hard rubber or vulcanite, or its equivalent, for holding artificial teeth, or teeth and gums, substantially as described." The specification does not mention any material to be used for forming the plate except soft rubber, or gum, componded with rubber, sulphur, etc,, "in the manner prescribed in the patent of Nelson Goodyear, dated May 6th, 1851, for making hard rubber, and is to be subjected to sufficient heat to harden it, substantially as directed in that Patent." "After the plate has been heated sufficiently to harden it, or convert it into hard rubber, or vulcanite so called, the mould is removed and the plate is polished ready for use."

Any vulcanized plate made from any vulcanizable compound would be comprised in the claim under words "hard rubber or vulcanite or its equivalent:" but no plate not vulcanizd or made of vulcanizable compound, would come within the claim of the Cummings patent.

Celluloid contains neither rubber nor any other vulcanizable gum, nor sulphur nor any other vulcanizing agent.

Plates made of Celluloid are neither vulcanized nor vulcanizable, and it is not true that the use of Celluloid is an infringement of the Cummings patent, Reissue No. 1,904, dated Mach 21st, 1865.

Referring to certain other letters also submitted to me, in which I find it stated that agents of the Goodyear Dental Vulcanite Company are demanding money and claiming license fees under this Cummings patent Reissue No. 1,904, for the past use of Celluloid, alleging that such use was an infringement of that patent, I would suggest that each dentist should protect himself against such unlawful exactions or attempts, by having any person enforcing such demand or claim arrested and dealt with by the proper local authorities, for obtaining money under false pretences.

Respectfully,

HENRY BALDWIN, JR., Counsellor at Law.

We ask the close attention of every one interested to the above communication.—ED.

### THE SMALL CATECHISM.

"Is Celluloid as now manipulated a success as a base for artificial dentures?"

Yes, as nearly so, as any of the cheap bases that are in use.

"Is it as good as RUBBER?" Yes in some respects better.

"Is it durable?"

Thus far it appears to be as good in this respect as any of the cheap bases.

"Can all dentists work it?"

Yes, when they know how. Many manipulate it with as good success as any thing else. Many complain that they can not work it.

Yes, and there is a still greater number who ought to weep that they can not work gold, continuous gum, porcelain etc.; indeed, do not know anything at all, practically, about these materials for artificial dentures, and yet they have no wail therefor.

"Has Celluloid any advantages over rubber?"

Yes, its color is greatly preferable, with it gum teeth are not requisite as a better imitation of the natural gums can be made with it than is usually made with porcelain gum of the rubber blocks. They can be more perfectly arranged and articulated than is possible with rubber sections of teeth.

"Has any improvement been make in Celluloid recently?"

Yes, a very marked improvement has been made within the last year and a half. It is constantly being improved, and this will continue till it is as nearly perfect as possible. Improvement is all the time being made in the mode of working it.

"Is it embarrassed by a patent?" It is not for dental purposes, no license is required for its use. It is not an infringement of any patent so far as we are aware. "Does Celluloid injure the mucous membrane with which it is in contact?"

We have seen no injurous effects from it. If it was half as bad as rubber in this respect we should discard it, and advise against its use.

### TENNESSEE DENTAL SOCIETY.

We had the privilege of being present at the recent meeting of this body, and we must say it was one of the best meetings we have attended for a long time. A larger number of papers, and good ones too, were read than is usual at our meetings, and they were, so far as time would admit, fully and freely discussed. The attendance was not very large, but those present went into the work with a will, demonstrating the fact that large numbers are not absolutely requisite to make an interesting and profitable meeting. The next number of the REGISTER will probably contain some of these papers.

### AMERICAN DENTAL ASSOCIATION.

The fifteenth annual meeting of the American Dental Association will be held at Niagara Falls, New York, commencing on the first Tuesday of August, 1875.

The committee have up to this date completed the following arrangements:

The Cataract House will accommodate delegates at $4.00 per day, and the International at $3.50. Rooms will be reserved at either house for parties making known their desires a few days beforehand. Gentlemen accompanied by their wives should state this fact when ordering rooms.

The N. Y. Central R. R. will sell round trip tickets from N. Y. to the Falls and return for $17.50.

The Great Western R. R. of Canada will sell tickets for the round trip at one full fare and a third. Parties desiring to avail themselves of these tickets should ask for excursion tickets to American Dental Association.

Round trip tickets from Chicago via Michigan Central R. R. will be sold at $20 which is less than one and a third fare.

The Grand Trunk R. R. will sell tickets for the round trip at one fare and a third.

The C., C., C. & I. R. R. will sell round trip tickets at the the rate of four cents a mile one way, to parties of twenty or more. C. STODDARD SMITH, Recording Secretary.
G. L. FIELD, Chairman Com. of Arrangements.

Round trip tickets will also be furnished to those going to the Association, via the Hamilton and Dayton, Dayton and Michigan and the Canada Southern R. R. for $16.00.

By the C., C., C. & I. R. R. parties leaving Cincinnati Monday morning, Aug. 2, at 7 o'clock will reach the Falls that night at 10 o'clock. And going by the Hamilton and Dayton and Canada Southern, leaving Cincinnati at the same time will arrive at the Falls next morning about 6 o'clock.

The Atlantic and Great Western R. R. will sell round trip tickets to the American Dental Association for $15.00.

### THE AMERICAN DENTAL CONVENTION.

The Committee have made arrangements with the proprietors of the Ocean House, Long Branch, for holding the Twenty-first Anniversary of the Convention at that place on the 10th of August, 1875, at 11 a. m.

The Messrs. Leland have, with their accustomed liberality, offered us a hall, free of charge, suitable for our meetings; also to deduct ten per cent from the bills of members stopping at their house. Ample arrangements have been made for the exhibition of instruments, implements, materials, etc. All manufacturers and venders are earnestly solicited to make the display as large and complete as possible.

The profession are cordially invited to be present at this our quarter centennial anniversary, believing it will be of unusual interest.

The Committee will secure rooms for those giving timely notice to the Chairman. J. G. Ambler, Chairman,
25 W. 23d st., New York.

### A NEW AUTOMATIC MALLET.

Invented and constructed by Dr. E. C. Hamilton, of Washington C. H., in this State, is soon to be introduced to the dental profession. It is simple in its construction, and definite and efficient in its action, and is not liable to become disarranged. From the slight examination we have made of it we think it equal, if not in some respects superior to any thing of the kind as yet presented.

It will very soon be in the market, and then each can judge for himself.

### NEW JERSEY STATE DENTAL SOCIETY.

The New Jersey State Dental Society meets at the Ocean House, Long Branch, on Tuesday, July 6th, and continues in session for two days. Let all who can attend.

C. S. Stockton, Newark, N. J.

